# Needle stick unjuries among health care workers – a report from India

**DOI:** 10.1186/1753-6561-5-S6-P225

**Published:** 2011-06-29

**Authors:** D Sureshkumar, V Ramasubramanian, K Abdulghafur

**Affiliations:** 1 Infectious disease, Apollo Hospitals, Chennai, India

## Introduction / objectives

Preventing needle stick injuries (NSIs) is a challenge faced in virtually every work environment. There are very few studies in India documenting frequencies and consequences of needle stick injuries (NSIs). We report a 30 months ongoing surveillance of NSIs happened in Apollo Hospitals, Chennai (large tertiary care hospital in India).

## Methods

Hospital infection control team document type of NSI, human immunodeficiency virus(HIV), Hepatitis B surface antigen (HBsAg, and hepatitis C virus (HCV) status of the source, anti HBs antibody titers of HCW, baseline and 6 months tests for HIV if the source was positive for HIV, and provided post-exposure prophylaxis to persons who had NSI.

## Results

Of the 118 needle stick injuries reported during the surveillance period 47 (40%) were nurses, 25 (21%) were lab technicians, 24 (20%) were doctors, 20 (17%) were housekeeping staff and 2(2%) were other staffs. Hollow bore needle constituted 80.1% (95) of the injuries, solid needles constituted 16.5% (19) of the injuries and other sharps constituted 3.4% (4) of the injuries. On source analysis 17, 9, and 8 were positive for HBsAg, HIV and HCV, respectively. Improper disposal of the needles (27%) & recapping of the needle (25.8%) were the predominant activities responsible for NSIs. 9 HCWs who sustained injury with HIV positive source were given immediate antiretroviral therapy for 4 weeks. Subsequent six-month follow-up showed zero seroconversion.

## Conclusion

NSIs were common among nurses & lab technicians and commonly take place in ICU. Half of the NSIs were happened after the usage of the needle before its disposal. Zero sero conversion for HIV was seen in NSIs with HIV positive source. Safer disposing methods are needed to reduce the incidence of NSIs.

## Disclosure of interest

None declared.

